# Autoallergy and what the Allergist Needs to know

**DOI:** 10.1007/s11882-025-01215-8

**Published:** 2025-08-09

**Authors:** Carolin Steinert, Margarita Pashuk, Pavel Kolkhir, Emek Kocatürk, Yi-Kui Xiang

**Affiliations:** 1 Institute of Allergology, Charité – Universitätsmedizin Berlin, corporate member of Freie Universität Berlin and Humboldt-Universität zu Berlin, Hindenburgdamm 27, 12203 Berlin, Germany; 2 Fraunhofer Institute for Translational Medicine and Pharmacology ITMP, Immunology and Allergology IA, Berlin, Germany; 3 Department of Biology, Chemistry and Pharmacy, Freie Universität Berlin, Berlin, Germany; 4 Department of Dermatology, Bahcesehir University School of Medicine, Istanbul, Turkey; 5 Shanghai Skin Disease Hospital, Tongji University School of Medicine, Shanghai, China

**Keywords:** Autoallergy, Atopic dermatitis, Asthma, Chronic spontaneous urticaria, IgE, Autoantibodies

## Abstract

**Purpose of the Review:**

This review aims to educate allergists about the concept of autoallergy by addressing five questions: 1) What is autoallergy and how does it differ from classical allergy? 2) How common is autoallergy? 3) Is autoallergy clinically relevant? 4) How can autoallergy be diagnosed? and 5) How is autoallergy treated?

**Recent Findings:**

In contrast to type I hypersensitivity against external allergens (allergy), autoallergy involves IgE autoantibodies targeting self-antigens. These are found in conditions like chronic spontaneous urticaria, atopic dermatitis, and asthma, with varying prevalence. While no standardized diagnostic tools exist, ELISA and basophil activation tests help identify the presence and function of IgE autoantibodies. Anti-IgE therapies have shown benefit, supporting their clinical relevance.

**Summary:**

Autoallergy is emerging as a distinct IgE-mediated mechanism that may contribute to chronic inflammation in immune-mediated diseases. Further investigation of this mechanism can improve disease stratification and enable more effective, targeted treatment strategies.

## Introduction

Asthma, atopic dermatitis (AD), Chronic urticaria (CU), and food allergies are among the most common immunologic disorders managed by allergists. Asthma is a respiratory disease of the lower airway characterized by variable symptoms such as cough, wheezing, shortness of breath and bronchial hyperreactivity. Several asthma endotypes have been described including Th2-high asthma, characterized by high levels of type 2 cytokines and eosinophilic airway inflammation and Th2-low (or non-Th2) asthma associated with neutrophilic or paucigranulocytic inflammation [1]. AD is a widespread, chronic inflammatory skin condition marked by itchy, red, and dry skin. The causes of AD are multifactorial including environmental triggers, genetic predisposition, a compromised skin barrier and immune system dysregulation [2]. As with asthma, distinct pathophysiological mechanisms exist for AD, however, defining of reproducible endotypes remain challenging [3]. Chronic urticaria, a mast cell-driven inflammatory skin disorder, is characterized by recurrent wheals, angioedema, or both, lasting for more than six weeks [4]. CU can be classified as chronic spontaneous urticaria (CSU) and chronic inducible urticaria (CIndU) with CSU occurring in the absence of identifiable physical or environmental stimuli. In recent years, advances in immunological research have revealed that autoimmunity plays a central role in the pathogenesis of CSU [5]. Food allergies are abnormal immune responses triggered by exposure to specific foods and are classically defined as immunoglobulin E (IgE)-mediated hypersensitivity reactions. However, recent advances, e.g. in diagnostic tools, have led to the proposal of distinct food allergy endotypes [6].

Affecting 30–40% of the global population [7], these conditions are becoming increasingly prevalent and complex, often involving multifactorial triggers and overlapping phenotypes. While traditionally viewed as disorders of hypersensitivity to exogenous antigens (allergy), emerging research has revealed that some diseases may also involve immune responses directed against self-antigens, a phenomenon termed autoallergy. This concept, situated at the intersection of allergy and autoimmunity, remains underrecognized in daily clinical practice. In this review, we aim to provide allergists with a concise and clinically relevant overview of autoallergy by addressing five key questions (Table 1): (1) What is autoallergy and how does it differ from classical allergy? (2) How common is autoallergy? (3) Is autoallergy clinically relevant? (4) How can autoallergy be diagnosed? and (5) How is autoallergy treated? In this review, we focus on asthma, AD, CU, and food allergy, while excluding conditions like systemic lupus erythematosus and bullous pemphigoid, where IgE autoantibodies have been described but are less central to allergy practice and have been discussed in a prior publication [8].

**Table 1:** Summary: Key questions and answers

Questions	Answers
What is autoallergy and how does it differ from classical allergy?	In autoallergy, the IgE antibodies are produced against internal proteins (autoantigens, endoallergens), and bind to surface receptors on mast cells and basophils, leading to their activation and disease symptoms. In contrast, traditional allergies involve IgE responses to external allergens like pollen or food proteins, although the symptom pattern is the same
How common is autoallergy?	Autoallergy is thought to be common, although its prevalence can vary greatly across different diseases, cohorts, and detection methods
Is autoallergy clinically relevant?	In some diseases such as AD and CSU, presence of IgE autoantibodies was associated with higher disease activity and comorbidities, although the evidence is not consistent and more research is needed
How can autoallergy be diagnosed?	Autoallergy can be assessed using several in vitro and in vivo methods designed to detect IgE autoantibodies and/or autoantigens, e.g. immunoassays, protein microarrays, functional assays (e.g. basophil activation test) and skin test. However, none of these methods are currently established in routine clinical practice for detection of autoallergy and the development of standardized, clinically validated diagnostic tools remains an urgent need
How is autoallergy treated?	Omalizumab, a monoclonal anti-IgE antibody, is approved for the treatment of asthma, CSU, food allergies. Other treatment options focus on inhibiting key cytokines involved in IgE production and mast cell activation, such as IL-4/IL-13 blockers (dupilumab), BTK inhibitors (remibrutinib), and anti-KIT antibodies which deplete mast cells (e.g., barzolvolimab)

### What is Autoallergy and how Does it Differ from Classical Allergy?

IgE is best known as a key effector in type I hypersensitivity reactions [9]. Classic IgE-mediated allergy involves sensitization to environmental (exogenous) antigens (e.g. pollen and food proteins), where antigen-presenting cells present these allergens to naive T cells. Differentiated Th2 cells activate B cells, initiating class-switch recombination and subsequent IgE production. IgE can bind to effector cells like basophils and mast cells via its high affinity receptor (FcεRI). Upon re-exposure, the allergen crosslinks surface-bound IgE on mast cells and basophils, triggering degranulation and release of histamine, leukotrienes, and cytokines [10].

Accumulating evidence has revealed that IgE, in addition to exogenous antigens, can also target self-antigens leading to autoimmune responses known as autoallergy, or type I autoimmunity [5]. In autoallergy, the immune system produces IgE autoantibodies directed against endogenous antigens also called autoantigens or autoallergens, including thyroid peroxidase (TPO), interleukin-24 (IL-24), manganese superoxide dismutase (MnSOD), among others [8]. As in classical allergy, this form of autoimmunity results in activation of mast cells and basophils through crosslinking of FcεRI-bound IgE, leading to inflammation and clinical manifestations such as wheals [5].

Endogenous IgE responses may arise through several immunological mechanisms that are increasingly recognized in chronic inflammatory and autoimmune conditions. One such mechanism is molecular mimicry, in which exogenous antigens (such as microbial or parasitic proteins) share structural or sequential similarities with tissue-specific human proteins or external allergens, leading to cross-reactive IgE responses^11^–^14^. For instance, Sánchez et al. proposed that TPO may resemble endogenous eosinophil proteins like eosinophil peroxidase, potentially triggering autoreactive IgE responses [12–14]. IgE reactivity to human profilin, the first IgE autoantigen to be identified, has been found in individuals with birch pollen allergy, suggesting molecular mimicry between plant and human homologs [15].

**Fig. 1:**
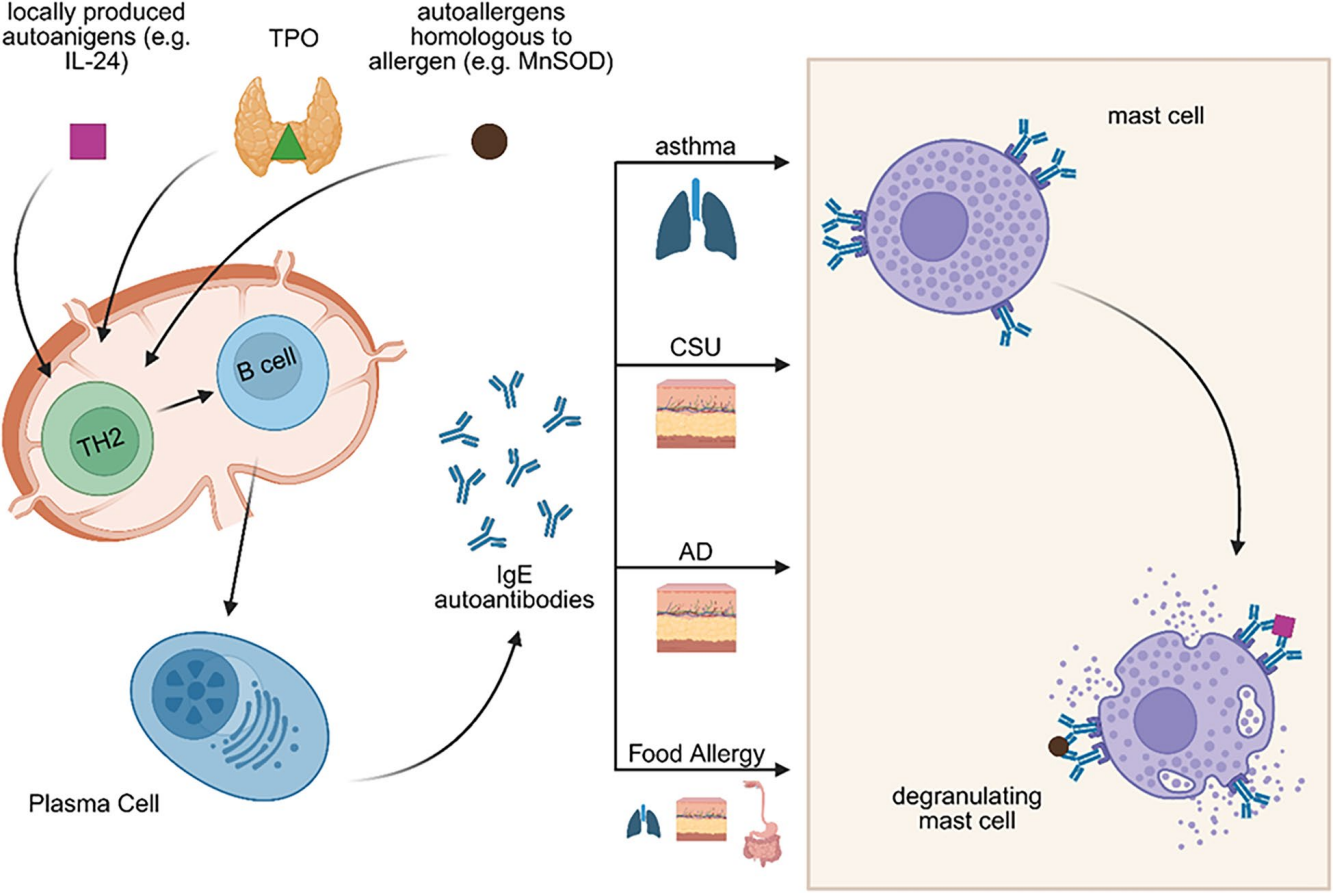
Pathogenesis of autoallergy in asthma, CSU, AD and Food Allergy. Similar to type I hypersensitivity reactions, autoallergy is also characterized by a two-step process consisting of a sensitization phase followed by an effector phase. However, the precise mechanisms underlying sensitization in autoallergy remain unknown. Several hypotheses have been proposed to explain the development of autoreactive IgE. One suggests that in the presence of alarmins, self-antigens may be taken up by antigen-presenting cells, leading to the activation of Th2 cells. These Th2 cells then promote the production of autoreactive IgE antibodies by plasma cells. Another proposed mechanism is molecular mimicry, in which structural similarities between environmental or microbial antigens and self-proteins lead to the production of IgE autoantibodies. IgE autoantibodies can bind to tissue-resident mast cells: in the lungs in asthma, in the skin in atopic dermatitis (AD), and in the lungs, intestines, and skin in food allergy. Mast cell activation and subsequent degranulation may be triggered by autoallergens that are cross-reactive with exoallergens (e.g. MnSOD), by re-exposure to locally produced autoallergens (e.g. IL-24), or by local allergens that cross-react with other human proteins (e.g. TPO and EXP). Created with BioRender.com

Another contributing mechanism is epitope spreading, where an initial immune response against a specific epitope gradually extends to involve other epitopes on the same or related self-antigens, further amplifying autoimmunity and autoallergy [12–14]. Additionally, local class-switch recombination to IgE may occur within tertiary lymphoid structures that develop in chronically inflamed tissues such as the skin. These ectopic lymphoid formations create a specialized microenvironment that supports ongoing B cell activation and class switching in situ, enabling the production of IgE against local tissue antigens independently of central immune organs [16, 17]. Such local IgE production has also been proposed in barrier organs, such as the respiratory epithelium and nasal mucosa, particularly in asthma and allergic rhinitis [18–21]. A proposed model of autoallergy (Fig. 1**)** mirrors the classical type I hypersensitivity cascade, with a sensitization phase followed by an effector phase, but with key differences in antigen origin.

Functional assays, such as the basophil activation test (BAT), and in vivo tests, e.g. skin prick tests and passive transfer experiments, underscore the contribution of autoreactive IgE to pathogenesis of asthma, AD, CSU, and food allergy. Notably, passive transfer of IgE against thyroid peroxidase (TPO) from CSU patients has been shown to induce wheal-and-flare reactions in healthy skin, as demonstrated in the study by Sanchez et al., further supporting the pathogenic role of autoreactive IgE in CSU [22]. Basophils from IgE anti-eosinophil cationic protein (ECP) and IgE anti-eosinophil peroxidase (EXP) positive asthmatic patients, for instance, showed a dose dependent activation after stimulation with the culprit autoantigen [13]. Similar, assays using rat basophilic leukemia cells demonstrated that human α-lactalbumin triggers mediator release in sensitized milk allergic individuals [23].

### How Common is Autoallergy?

Autoallergy has been detected in a range of immune-mediated disorders, yet the prevalence of autoreactive IgE varies substantially depending on the underlying disease, the specific autoallergen, the characteristics of the study population, and the detection methods employed. Table 2 provides an overview of IgE autoreactivity across the reported disease entities, including information on identified autoantigens, prevalence, and detection techniques.

**Table 2:** Prevalence of IgE autoantibodies in atopic diseases

Disease	Autoantigen	Prevalence disease (%)	Prevalence HC (%)	Method	Reference
Asthma	Periplakin	8.7	n.a	Western Blot	Taillé et al. [25]
	Rheumatoid factor	70	n.a	ELISA	Gioud-Paquet et al. [26]
	Platelet antigens	n.a	n.a	Western Blot	Lassalle et al. [27]
	EXP	14.5	3.3	ELISA	Sánchez et al. [13]
	ECP	9	5	ELISA	Sánchez et al. [13]
	DFS70	16	n.a	Western Blot	Ochs et al. [24]
Allergic bronchopulmonary aspergillosis	Acidic ribosomal phosphoprotein type 2	n.a	n.a	ELISA, Immunoblots	Mayer et al. [59]
	MnSOD	n.a	n.a	ELISA	Crameri et la. [86]
	Thioredoxin	around 50	n.a	ELISA	Glaser et al. [60]
Atopic dermatitis	Actin α	15.5	0	ELISA	Zeller et al. [11]
	Autologous Sweat	84.4	11.1	Skin testing	Hide et al. [40]
		77	8.7	Basophil histamine release	Tanaka et al. [36]
	Squamous cell antigen recognized by T cells (SART) 2	2.5	4.9	ELISA	Kawamoto et al. [87]
	SART 3	START 3 (VYDYNCHVDL): 37.5 START 3 (AYIDFEMKI): 15	START 3 (VYDYNCHVDL): 29.3 START 3 (AYIDFEMKI): 17.1	ELISA	Kawamoto et al. [87]
	Adenocarcinoma antigensrecognized by T cells 4	10	12.2	ELISA	Kawamoto et al. [87]
	Cyclophilin B	9.2	0	ELISA	Zeller et al.[11]
		Cyclophilin B peptide (KFHRVIKDF): 27.5 Cyclophilin B peptide (DFMIQGGD): 17.5	Cyclophilin B peptide (KFHRVIKDF): 29.3 Cyclophilin B peptide (DFMIQGGD): 31.7	ELISA	Kawamoto et al. [87]
	Dense fine speckels/lens epithelium derived growth factor DFS70/LEDGF	14.8	0	ELISA	Wantanabe et al. [34]
		30	n.a	Western Blot	Ochs et al. [24]
	ECP	26.6	1.6	ELISA	Sanchez et al. [14]
	elF6	25.4	0	ELISA	Zeller et al.[11]
	Epidermal keratinocytes	37	0	Immunoblot	Kortekangas-Savolainen et al. [39]
	Epidermis	23.4	0	Western Blot	Altrichter at el. [41]
	Epithelial cell line A431	21.8	0	Western Blot	Altrichter at el. [41]
		43.1	n.a	Western Blot	Natter et al. [37]
		50	0	Western Blot	Valenta et al. [35]
		23	0	Western Blot	Mothes et al. [38]
		72.7	0	Western Blot	Mittermann et al. [88]
	EXP	28.8	3.3	ELISA	Sanchez et al.[14]
	HLA-DR	8.7	0	ELISA	Zeller et al.[11]
	Hom s 1	n.a	n.a	Immunoblot	Valenta et al. [42]
	Hom s 2	72.7	0	Western Blot	Mittermann et al. [88]
		27.3	0	Western Blot	Mittermann et al. [88]
	Hom s 4	10	n.a	Immunoblot	Aichberger et al. [43]
	MnSOD	36	n.a	ELISA	Schmid-Grendelmeier et al. [44]
		14.8	n.a	Skin Prick Test	Guarneri et al. [58]
		75	0	ELISA	Andersson et al. [89]
	Thioredoxin	23.4	0	ELISA	Zeller et al. [11]
		5	0	ELISA	Wantanabe et al. [34]
	RP 1	29	0	ELISA	Zeller et al. [11]
	Tubulin α	21.7	0	ELISA	Zeller et al. [11]
Birch pollen allergy	Profilin	n.a	10–30	Immunoblots	Valenta et al. [15]
Chronic spontaneous urticaria	Double-stranded DNA (dsDNA)	n.a	n.a	Indirect ELISA	Hatada et al. [90]
	Eosinophil cationic protein	5.4	1.6	Direct ELISA after IgG depletion	Sánchez et al. [14]
	Eosinophil Peroxidase	10.9	3.3	Direct ELISA after IgG depletion	Sánchez et al. [14]
	FcεRI	30	n.a	Sandwich ELISA	Asero et al. [91]
	Interleukin 24 (IL-24)	80	20	Microarray and Sandwich ELISA	Schmetzer et al. [56]
		31	n.a	Site direct ELISA	Xiang et al. [50]
	Thyroglobulin (TG)	5	n.a	Modified commercial ELISA	Concha et al. [92]
		7	2.9	ELISA kit	Zhang et al. [52]
		35	25	Sandwich ELISA	Asero et al. [91]
		87	n.a	Sandwich ELISA	Cugno et al. [70]
	Thyroid peroxidase (TPO)	54	20	Site-directed human IgE capture ELISA	Altrichter et al. [53]
		7.5	0	Direct ELISA	Shin et al. [93]
		27.2	5.5	Direct ELISA after IgG depletion	Sánchez et al. [14]
		34	8.1	Direct ELISA after IgG depletion	Sánchez et al.[22]
		5	n.a	Modified commercial ELISA	Concha et al. [92]
		18	6.8	ELISA kit	Zhang et al.[52]
		43	n.a	Direct ELISA after IgG depletion	Sánchez et al. [51]
		100	n.a	Side direct ELISA	Çildağ et al. [94]
		0	n.a	Modified commercial radioimmunoassay	Tedeschi et al. [95]
		41	n.a	Side direct ELISA	Xiang et al. [50]
	Tissue Factor (TF)	50	36	Sandwich ELISA	Asero et al. [91]
		60	n.a	Sandwich ELISA	Cugno et al. [70]
	Tissue Transglutaminase 2	20.6	13	Capture ELISA	Su et al. [49]
Milk allergy	α-Lactalbumin	100	n.a	ELISA	Maynard et al. [28]
	Peptides of α-lactalbumin, β-casein, and κ-casein	60	n.a	Immunoblot	Järvinen et al. [23]
	β-casein	35	n.a	ELISA	Bernard et al. [29]
	Human milk	100	0	Western Blot	Cantisani et al. [30]

n.a.: not available, HC: healthy controls

Studies on asthma have identified IgE autoantibodies targeting various autoantigens, including DFS70 [24], periplakin [25], rheumatoid factor [26], platelet antigens [27], EXP and ECP [13] with reported prevalence rates ranging from 8.7% to 70%. In the context of food allergy, existing reports on IgE autoantibodies are limited to cow’s milk allergy, where autoreactive IgE has been detected against human homologs of α-lactalbumin, β-casein, and κ-casein (see Table 2). Notably, the prevalence of autoreactive IgE in milk allergy appears to be high, ranging from 35% to 100% [23, 28–30].

The concept of autoimmunity in AD has been recognized for several decades, with studies dating back over eighty years identifying autoreactivity to human dander in patients with eczema [31, 32]. Thereafter, multiple studies [24, 33–41] have confirmed the presence of autoreactive IgE in individuals with AD, with at least 140 [11] different self-antigens identified. Examples of human autoantigens in AD include autologous sweat, Homo sapiens allergen 1–5 [42, 43], and MnSOD [44]. Prevalence studies on autoreactive IgE in AD patients have shown a broad range, from 23% to as high as 91% [8], depending on the sample size and methodology. Larger-scale research typically reported prevalence rates around 20–30% [8]. Of note, a recent analysis found autoreactive IgE in 16.4% of those with both AD and other atopic conditions and only 9.6% in those with only AD [45].

In CSU, several IgE autoantibody targets have been identified, including IL-24, TPO, thyroglobulin (TG), double-stranded DNA (dsDNA), ECP, EXP, FcεRI, tissue factor (TF) and tissue-transglutaminase 2 [46–49, 96]. Reported prevalence rates of autoreactive IgE in CSU vary considerably and range from 0–100% [8]. However, the majority of studies report a prevalence between 30–60% [8, 48]. A recent study involving 111 CSU patients showed that more than half of the patients (58%) exhibited an autoallergic phenotype, suggesting that autoallergy is a common feature of CSU [50].

Autoreactive IgE can also be detected in some healthy individuals, with reported prevalence rates ranging from 0 to ~30% (see Table 2). However, in these cases, such antibodies are generally regarded as non-pathogenic. For instance, Sánchez et al. demonstrated that healthy subjects with detectable IgE against ECP and EXP showed minimal to no basophil activation upon stimulation with these autoantigens [13, 14].

### Is Autoallergy Clinically Relevant?

The clinical significance of autoallergy is based on the evidence linking autoreactive IgE with disease severity and other clinical features. In AD, IgE autoantibodies have been also linked to dry skin, dyshidrosis, itchyosis, infections and pruritus [38]. In asthma, patients with IgE anti-periplakin had a higher frequency of nasal polyps than patients without autoantibodies [25]. In CSU, IgE anti-TPO has been associated with higher frequency of atopy and asthma [51]. Additionally, IgE anti-TPO levels correlated with IgG anti-TPO levels [52, 53] supporting the notion that CSU patients are at risk for autoimmune thyroid diseases and thyroid dysfunction [5]. Cross-reactivity between bovine and human proteins is particularly clinically relevant when infants with cow’s milk allergy remain symptomatic despite maternal avoidance of dairy [23, 54, 55].

In CSU, IgE autoantibody levels were associated with disease activity in some studies [50, 56], but not in others [22, 52, 53]. For example, IgE anti-IL-24 levels correlated with the urticaria activity score over 7 days (UAS7) [56]. Further, it has been shown that autoantibodies are elevated during exacerbation periods [51]. Also, in AD autoreactive IgE correlates with disease severity [37, 38, 41, 44], while some studies failed to find this correlations [57]. For instance, AD patients exhibiting IgE autoreactivity against epidermal proteins had higher Investigator’s Global Assessment (IGA) and Eczema Area and Severity Index (EASI) scores compared to AD patients without autoreactivity [41]. Also, most of the asthma patients exhibiting autoantibodies against EXP and/or ECP had severe asthma [13].

### How Can Autoallergy be Diagnosed?

In classical allergy, there are several certified and approved assays for the detection of allergen specific IgE. In contrast, currently there is no approved medical test for the determination of IgE autoantibodies in routine clinical practice. As of yet, all existing assays for the assessment of serum IgE autoantibody levels, e.g. immunoassays such as ELISA or Western Blot, are used for research purposes (Table 2). Reported prevalence rates vary considerably, e.g. 0–100% for IgE anti-TPO in CSU, due to a lack of standardization, selection bias, small sample sizes, and methodological limitations. In some rare cases also, skin prick testing has been used to identify the presence of autoreactive IgE [40, 58–60]. Of note, recently the usage of allergen-specific IgE microarrays has been proposed for its usage in AD and other autoallergic diseases. In particular, the measurement of IgE to human-homologs exogenous molecular allergens might be a potential biomarker for autoallergy [61].

BAT and basophil histamine release assay (BHRA) are used to demonstrate functional relevance of autoantigens and/or as diagnostic tools, e.g. in food allergy [62, 63]. Various BAT protocols are available, broadly categorized into direct and indirect approaches. In the direct method, fresh whole blood from the patient is used, preserving the in vivo IgE environment. In contrast, the indirect approach involves sensitizing donor basophils with serum from the patient, thereby transferring the relevant IgE antibodies. Following antigen stimulation, activation markers such as CD203c and CD63 are typically measured using flow cytometry to quantify basophil activation. BHRA is a similar technique but, instead of measuring activation markers on the cell surface, it measures the release of histamine.

Further efforts are needed to develop validated and reproducible tools that can reliably detect and quantify autoreactive IgE. Establishing clinical thresholds and correlating IgE autoreactivity with disease activity and treatment response will be critical steps toward integrating autoallergy diagnostics into routine practice. Integration of high-throughput platforms, such as multiplex proteomics, could also provide new insights and support biomarker discovery for precision therapy in the field of autoallergy.

### How is Autoallergy Treated?

The main therapeutic strategy in treating autoallergy is to neutralize circulating IgE and thereby disrupting the downstream activation cascade of effector cells, such as mast cells. Omalizumab remains the only anti-IgE monoclonal antibody currently approved for allergic asthma, CSU, and food allergy. Furthermore, several omalizumab biosimilars are licensed or will be licensed for these indications soon [64].

Mechanistically, omalizumab binds to a structural epitope within the Cε3 domain of IgE, a region critical for the interactions with both, FcεRI and the low-affinity IgE receptor (CD23). By targeting this domain, omalizumab sequesters free IgE and prevents its binding to receptors on mast cells, basophils, B cells and antigen-presenting cells [65]. Importantly, omalizumab does not bind IgE that is already attached to FcεRI, thereby avoiding immediate cell activation [66]. The reduction of free circulating IgE leads to several immunomodulatory effects, including downregulation of FcεRI expression on mast cells, basophils, and dendritic cells, resulting in decreased sensitivity to antigens and reduced cell activation [5]. Additionally, omalizumab attenuates IgE-facilitated antigen presentation by dendritic cells [67].

The rapid and favorable response to omalizumab points to the clinical relevance of autoreactive IgE in CSU. Omalizumab led to complete response to treatment in 70% of CSU patients with IgE anti-TPO [68]. Although this trial did not investigate whether patients with IgE autoantibodies can better respond to omalizumab than those without, a recent study in bullous pemphigoid, another skin disease with IgE autoantibodies, supported this notion. In particular, 75% of patients with bullous pemphigoid with increased serum baseline level of IgE anti-BP180-NC16A showed completed response to omalizumab as compared to 41% of those who did not have these autoantibodies (p = 0.01) [69]. Preliminary evidence suggests that treatment with omalizumab can significantly reduce serum levels of IgE autoantibodies, such as IgE anti–TF and IgE anti–TG, in CSU patients as early as one week of treatment [70].

In addition to directly targeting IgE itself, patients with autoallergic diseases may benefit from addressing other key pathogenic factors, including the cytokines IL-4 and IL-13, Bruton Tyrosine Kinase (BTK) and KIT receptor. Dupilumab, an IL-4Rα inhibitor that blocks both IL-4 and IL-13 signaling, is approved for asthma, AD, and CSU (in US, Japan, Brazil and the United Arab Emirates and is currently under review in the EU). Dupilumab has been shown to significantly reduce total IgE levels in AD patients [71, 72], as well as reductions of up to 86% in specific IgE levels against food allergens after three years of treatment [73]. BTK plays a critical role in both B cell receptor (BCR) signaling and FcεRI-mediated mast cell activation [74–77]. Currently, several BTK inhibitors, including remibrutinib, rilzabrutinib, atuzabrutinib, branebrutinib, and fenebrutinib, are undergoing clinical evaluation for potential use in food allergy, CSU, asthma, and AD. Remibrutinib, for instance, has demonstrated both efficacy and a favorable safety profile in the treatment of CSU [78, 79], as it effectively inhibits the activation of mast cells and basophils triggered by IgE cross-linking or serum-derived activating factors [80]. Given its ability to block IgE-mediated cell degranulation, remibrutinib may also be beneficial in autoallergic conditions [81]. Additionally, BTK inhibition may limit autoreactive B cell development and promote self-tolerance by preventing bypass of peripheral tolerance checkpoints [82]. The KIT receptor, a key regulator of mast cell survival, proliferation, and activation, can be blocked by monoclonal antibodies such as barzolvolimab. In a phase 1b trial, 43% of barzolvolimab-treated CSU patients achieved a complete response (UCT = 16) by week 12 [83], demonstrating the clinical potential of mast cell-depleting therapies for autoallergy. Phase 2 and 3 clinical trials of barzolvolimab in CSU are ongoing.

## Conclusion

Several unmet needs hinder the recognition of autoallergy in clinical practice, including the lack of standardized, clinically validated assays, absence of biomarkers for autoallergy or treatment response, and a need for disease-modifying therapies that target the underlying drivers of the disease rather than symptoms [84]. Studies investigating both disease-specific and cross-disease aspects of autoallergy, are needed. Recent research initiatives such as European Network for IgE-Mediated Autoimmunity and Autoallergy (ENIGMA) [47] and projects, e.g. CU-TIGER (The characterization of urticaria markers including anti-TPO and IgE in serum) [85], will address these gaps, including identification of the novel autoantigens and corresponding IgE autoantibodies, investigation of their clinical relevance and standardization of assay protocols in autoallergy research [47]. These efforts are expected to advance our mechanistic understanding of autoallergy, facilitate the implementation of assays in clinical practice, and guide the development of targeted, durable therapies for autoallergic diseases.

## Key References

- Kortekaas Krohn I, Badloe FMS, Herrmann N, Maintz L, De Vriese S, Ring J, et al. Immunoglobulin E autoantibodies in atopic dermatitis associate with Type-2 comorbidities and the atopic march. Allergy. 2023;78(12):3178–92. 10.1111/all.15822.
  - This large-scale study on over 670 serum samples shows that IgE autoantibodies are more prevalent in AD patients with Type 2 comorbidities (asthma, allergic rhinitis, food allergy). It also links IgE autoreactivity with younger age and environmental factors, and suggests its potential role as a predictive biomarker for the atopic march.
- **Ref Nr. 47**Kolkhir P, Altrichter S, Badloe FMS, Belasri H, Charles N, De Vriese S, et al. The European Network for IgE-Mediated Autoimmunity and Autoallergy (ENIGMA) initiative. Nat Med. 2024;30(4):920–2. 10.1038/s41591-024-02819-9.
  - This paper introduces ENIGMA, a European collaborative research initiative focused on IgE-mediated autoimmunity and autoallergy. It highlights the urgent need for standardized diagnostic tools and deeper mechanistic understanding to advance clinical care for allergic and autoallergic diseases.
- **Ref Nr. 50**Xiang YK, Kolkhir P, Scheffel J, Sauer M, Vera C, Frischbutter S, et al. Most Patients With Autoimmune Chronic Spontaneous Urticaria Also Have Autoallergic Urticaria, but Not ViceVersa. J Allergy Clin Immunol Pract. 2023;11(8):2417–25 e1. 10.1016/j.jaip.2023.02.006.
  - This study provides key evidence that autoallergic urticaria (driven by IgE autoantibodies) is more common than autoimmune urticaria (driven by IgG autoantibodies) in CSU patients. It underscores the clinical relevance of overlapping IgE and IgG autoimmunity and calls for endotype-based stratification in diagnosis and treatment.

## Data Availability

No datasets were generated or analysed during the current study.

## Abbreviations

AD: Atopic Dermatitis
ASST: Autologous Serum Skin Test
ABPA: Allergic Bronchopulmonary Aspergillosis
BTK: Bruton's Tyrosine Kinase
BAT: Basophil Activation Test
BHRA: Basophil Histamine Release Assay
BCR: B cell Receptor
CU: Chronic Urticaria
CSU: Chronic Spontaneous Urticaria
CIndU: Chronic Inducible Urticaria
dsDNA: Double-stranded DNA
IL-24: Interleukin-24
IgE: Immunoglobulin E
JAK: Janus Kinase
MAT: Mast Cell Activation Test
MC: Mast Cell
MRGPRX2: Mas-related G Protein-coupled Receptor X2
SCF: Stem Cell Factor
sIgE: Specific IgE
TG: Thyroglobulin
TF: Tissue Factor
TPO: Thyroid Peroxidase
TSLP: Thymic Stromal Lymphopoietin
UAS: Urticaria Activity Score
